# PAP and NT5E inhibit nociceptive neurotransmission by rapidly hydrolyzing nucleotides to adenosine

**DOI:** 10.1186/1744-8069-7-80

**Published:** 2011-10-19

**Authors:** Sarah E Street, Paul L Walsh, Nathaniel A Sowa, Bonnie Taylor-Blake, Thomas S Guillot, Pirkko Vihko, R Mark Wightman, Mark J Zylka

**Affiliations:** 1 Department of Cell and Molecular Physiology, University of North Carolina, CB #7545, Chapel Hill, North Carolina, 27599, USA; 2 Department of Chemistry, University of North Carolina, CB #3290, Chapel Hill, North Carolina, 27599, USA; 3 UNC Neuroscience Center, University of North Carolina, CB #7250, Chapel Hill, North Carolina, 27599, USA; 4 Department of Clinical Medicine, Division of Clinical Chemistry, HUSLAB, P.O. Box 62, FI-00014, University of Helsinki, Finland

**Keywords:** pain, nociception, ectonucleotidase, adenosine, fast-scan cyclic voltammetry, field recordings

## Abstract

**Background:**

Prostatic acid phosphatase (PAP) and ecto-5'-nucleotidase (NT5E, CD73) produce extracellular adenosine from the nucleotide AMP in spinal nociceptive (pain-sensing) circuits; however, it is currently unknown if these are the main ectonucleotidases that generate adenosine or how rapidly they generate adenosine.

**Results:**

We found that AMP hydrolysis, when measured histochemically, was nearly abolished in dorsal root ganglia (DRG) neurons and lamina II of spinal cord from *Pap/Nt5e *double knockout (dKO) mice. Likewise, the antinociceptive effects of AMP, when combined with nucleoside transport inhibitors (dipyridamole or 5-iodotubericidin), were reduced by 80-100% in dKO mice. In addition, we used fast scan cyclic voltammetry (FSCV) to measure adenosine production at subsecond resolution within lamina II. Adenosine was maximally produced within seconds from AMP in wild-type (WT) mice but production was reduced >50% in dKO mice, indicating PAP and NT5E rapidly generate adenosine in lamina II. Unexpectedly, we also detected spontaneous low frequency adenosine transients in lamina II with FSCV. Adenosine transients were of short duration (<2 s) and were reduced (>60%) in frequency in *Pap*^*-/-*^, *Nt5e*^*-/-*^ and dKO mice, suggesting these ectonucleotidases rapidly hydrolyze endogenously released nucleotides to adenosine. Field potential recordings in lamina II and behavioral studies indicate that adenosine made by these enzymes acts through the adenosine A_1_ receptor to inhibit excitatory neurotransmission and nociception.

**Conclusions:**

Collectively, our experiments indicate that PAP and NT5E are the main ectonucleotidases that generate adenosine in nociceptive circuits and indicate these enzymes transform pulsatile or sustained nucleotide release into an inhibitory adenosinergic signal.

## Background

Neurons and glia throughout the nervous system release adenosine 5'-triphosphate (ATP) spontaneously and in response to diverse pathological insults, including tissue damage, inflammation, hypoxia and nerve injury [1-4]. In the somatosensory system, ATP excites and sensitizes nociceptive DRG neurons and can activate spinal microglia [5]. The excitatory effects of extracellular ATP can be terminated by a cascade of ectonucleotidases that hydrolyze ATP to adenosine [6,7]. The speed at which these ectonucleotidases work in any region of the nervous system is unknown. Adenosine can signal through the adenosine A_1_ receptor (A_1_R) to inhibit neurotransmission and nociception [8,9].

Recently, we identified two ectonucleotidases in nociceptive neurons that hydrolyze extracellular AMP to adenosine. These enzymes include the transmembrane isoform of prostatic acid phosphatase (PAP, also known as ACPP) and ecto-5'-nucleotidase (NT5E). PAP and NT5E are extensively co-localized in nociceptive DRG neurons and on axon terminals located in lamina II of the dorsal spinal cord [10,11]. PAP deficient (*Pap*^-/-^) and NT5E deficient (*Nt5e*^-/-^) mice have similar behavioral phenotypes, including enhanced nociceptive sensitization following nerve injury and following peripheral inflammation. Mice lacking A_1_R likewise show enhanced sensitization in models of chronic pain [12]. Humans with null mutations in *Nt5e *develop very painful arterial calcifications [13], suggesting loss of this adenosine-generating ectonucleotidase exacerbates an already painful condition in humans [14]. While these and other data suggest adenosine tonically inhibits nociceptive circuits [15,16], the time frame over which adenosine is produced and the molecular source of adenosine (from ectonucleotidases and/or directly released from cells) are unknown [17].

Several studies suggest ectonucleotidases inhibit nociception by generating adenosine from endogenously released nucleotides. Notably, pharmacological inhibitors of ectonucleotidases reduce adenosine production from AMP in dorsal spinal cord and reduce adenosine release in dorsal spinal cord synaptosomes [18,19]. Moreover, AMP hydrolysis in spinal lamina II was reduced, but not eliminated, in *Pap*^*-/-*^ and *Nt5e*^*-/-*^ single knockout mice [10,11]. Lastly, intrathecal injection of soluble (non-membrane bound) PAP or NT5E protein had long-lasting (2-3 days) antinociceptive effects that were entirely dependent on A_1_R activation [11,20-22].

Here, we generated mice lacking PAP and NT5E to investigate the combined importance of these enzymes in nociceptive mechanisms. As part of this study, we used FSCV to measure adenosine levels at subsecond resolution in the spinal microdomain (lamina II) where these enzymes are located [23,24]. Our data indicate PAP and NT5E rapidly hydrolyze nucleotides to adenosine and generate inhibitory adenosine transients in spinal nociceptive circuits.

## Results

### PAP and NT5E are the main AMP ectonucleotidases in spinal nociceptive circuits

**Figure 1:**
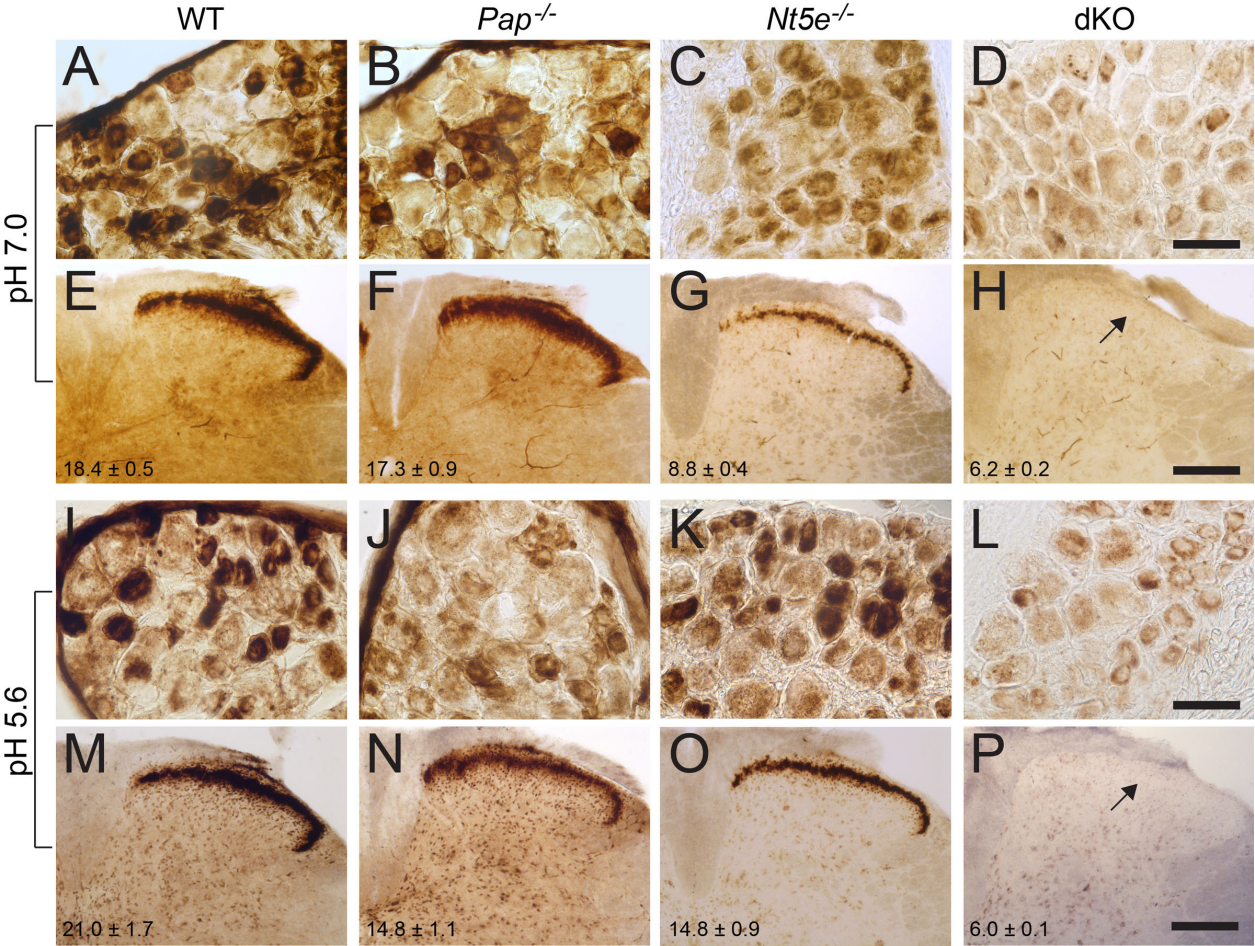
**AMP hydrolytic activity is reduced in DRG and spinal cord of *Pap***^***-/-***^***, Nt5e***^***-/-***^***, *and dKO mice**. Lumbar (A-D, I-L) DRG and (E-H, M-P) spinal cord sections from WT, *Pap*^*-/-*^*, Nt5e*^*-/-*^*, *and dKO mice were stained using AMP histochemistry at pH 7.0 or pH 5.6. Arrows (H and P) point to lamina II. Staining intensity in lamina II was quantified from 7-10 sections per genotype as described (Methods) and reported (bottom left) as means ± s.e.m. Repeated measures one-way ANOVA and Bonferroni's post-hoc tests were used to compare staining intensity between genotypes. At pH 7.0, staining intensity from *Nt5e*^*-/-*^ and dKO slices were significantly decreased from WT (*P *< 0.0005), and at pH 5.6, *Pap*^*-/-*^*, Nt5e*^*-/-*^ and dKO were all significantly decreased from WT (*Pap*^*-/-*^ and *Nt5e*^*-/-*^, *P *< 0.05; dKO, *P *< 0.0005). AMP concentration was 1 mM for DRG and 6 mM for spinal cord sections. Similar results were obtained from n = 3 mice of each genotype. Scale bars: 50 μm (D and L), 200 μm (H and P).

Using enzyme histochemistry, we previously found that AMP hydrolysis was reduced in DRG neurons and in spinal lamina II of *Pap*^*-/-*^ mice (at pH 5.6) and *Nt5e*^-/-^ mice (at pH 7.0) [10,11]. Since AMP hydrolysis was not eliminated in either single knockout under any experimental condition, this suggested both enzymes might contribute additively to AMP hydrolysis. To test this hypothesis, we bred *Pap*^*-/-*^ and *Nt5e*^*-/-*^ mice to generate dKO mice. dKO mice were viable, had average sized litters and had no obvious physical abnormalities. We then stained lumbar DRG and spinal cord sections from WT, *Pap*^*-/-*^*, Nt5e*^*-/-*^*, *and dKO mice using AMP enzyme histochemistry at neutral and acidic pH (Figure 1). Staining intensity in lamina II was quantified through image analysis. We found that small to medium diameter DRG neurons, the epineurium surrounding DRG, and nociceptive axon terminals in lamina II were intensely stained in tissues taken from WT mice (Figure 1A, E, I, M). Staining was noticeably decreased in DRG neurons and lamina II from *Pap*^*-/-*^ mice at pH 5.6 (Figure 1J, N) and *Nt5e*^*-/-*^ mice at pH 7.0 (Figure 1C, G), while epineurium staining was eliminated in *Nt5e*^-/-^ mice at both pHs (Figure 1C, K). Strikingly, staining of dKO DRG neurons was reduced at both pHs (Figure 1D, L) and nociceptive axon terminal staining in lamina II was eliminated at both pHs in dKO mice (Figure 1H, P). These findings suggest that PAP and NT5E are the main enzymes that hydrolyze extracellular AMP in DRG to spinal nociceptive circuits.

**Figure 2:**
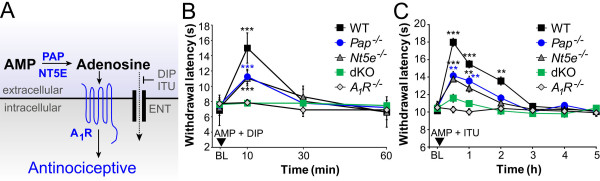
**The A**_**1**_**R dependent thermal antinociceptive effect of AMP, when combined with nucleoside transport inhibitors, is reduced in *Pap***^***-/-***^***, Nt5e***^***-/-***^***, *and dKO mice**. (A) AMP is hydrolyzed to adenosine by PAP and NT5E. Since adenosine is rapidly metabolized and transported into cells, we found it was necessary to block the reuptake of adenosine with nucleoside transport (ENT) inhibitors (like ITU or DIP). This allowed us to detect antinociceptive effects of adenosine under experimental conditions (behavior, field recordings). (B) Noxious thermal sensitivity was measured before (baseline, BL) and after i.t. injection of AMP (200 nmol)+DIP (5 nmol). These drugs have no antinociceptive effects when injected individually at these concentrations (not shown). A repeated measures two-way ANOVA was performed to compare all data sets (*P *< 0.0005 for WT, *Pap*^*-/-*^ and *Nt5e*^*-/-*^), and Bonferroni's post-hoc tests were performed to compare each genotype to baseline values at each time point. (n = 8 mice per genotype). (C) Noxious thermal sensitivity was measured before (BL) and after i.t. injection of AMP (200 nmol)+ITU (5 nmol). These drugs have no antinociceptive effects when injected individually at these concentrations [10]. A repeated measures two-way ANOVA was performed to compare data sets (*P *< 0.0005 for WT, *Pap*^*-/-*^, and *Nt5e*^*-/-*^), and Bonferroni's post hoc-tests were performed to compare each genotype to baseline at each time point. (n = 9-10 mice per genotype). *A*_*1*_*R*^*-/-*^ data in (C) replotted from [10]. (C-E) All data are presented as means ± s.e.m. *P < 0.05, **P < 0.005, ***P < 0.0005.

While these histochemical experiments at different pHs allowed us to discriminate staining for PAP and NT5E, a pH value of 5.6 may have limited physiological relevance (although see Discussion). Thus, we next sought to determine if PAP and NT5E were the main AMP hydrolytic enzymes *in vivo*, using a behavioral readout that is dependent on production of adenosine. To accomplish this, we intrathecally (i.t.) injected mice with AMP and dipyridamole (DIP) then measured adenosine receptor-dependent changes in noxious thermal sensitivity (Figure 2A). DIP is a nucleoside transport inhibitor that allows extracellular adenosine to build up and hence more effectively signal through adenosine receptors [25]. When AMP (200 nmol) and DIP (5 nmol) were injected (i.t.) individually, neither drug altered noxious thermal sensitivity in WT mice at 10, 30 or 1 hr post injection (data not shown). However, when combined, AMP+DIP had a brief (10 min) but significant thermal antinociceptive effect in WT mice (Figure 2B). This thermal antinociceptive effect was reduced by 54.7 ± 18.4% in *Pap*^*-/-*^ mice, 44.2 ± 21.0% in *Nt5e*^*-/-*^ mice and eliminated (not significantly different from baseline) in dKO mice. Moreover, AMP+DIP had no antinociceptive effect in *A*_*1*_*R*^*-/-*^ mice, indicating this behavioral effect was dependent on A_1_R activation.

To determine if these *in vivo *results could be reproduced with a different transport inhibitor, we co-injected mice with AMP and 5-iodotubericidin (ITU) (Figure 2A)--a drug that inhibits adenosine kinase and nucleoside transporters at equivalent concentrations [26]. AMP alone and ITU alone had no effect on thermal sensitivity in mice [10]. However, these drugs (AMP+ITU) had a 2 hr thermal antinociceptive effect when co-injected (Figure 2C), consistent with a previous study [10]. In addition, we found that the thermal antinociceptive effect of AMP+ITU was reduced by 57.8 ± 8.2% (percentage based on quantifying the area under the curve relative to WT mice) in *Nt5e*^-/-^ mice, 45.9 ± 8.0% in *Pap*^*-/-*^ mice and by 83.0 ± 5.1% in dKO mice (Figure 2C). We previously found that the thermal antinociceptive effect caused by AMP+ITU were fully dependent on A_1_R activation [10]. Taken together, these data with two different nucleoside transport inhibitors provide compelling evidence that PAP and NT5E account for the majority of all AMP hydrolytic activity in spinal nociceptive circuits under physiologically relevant *in vivo *conditions.

### PAP and NT5E rapidly generate adenosine from AMP in spinal lamina II

**Figure 3:**
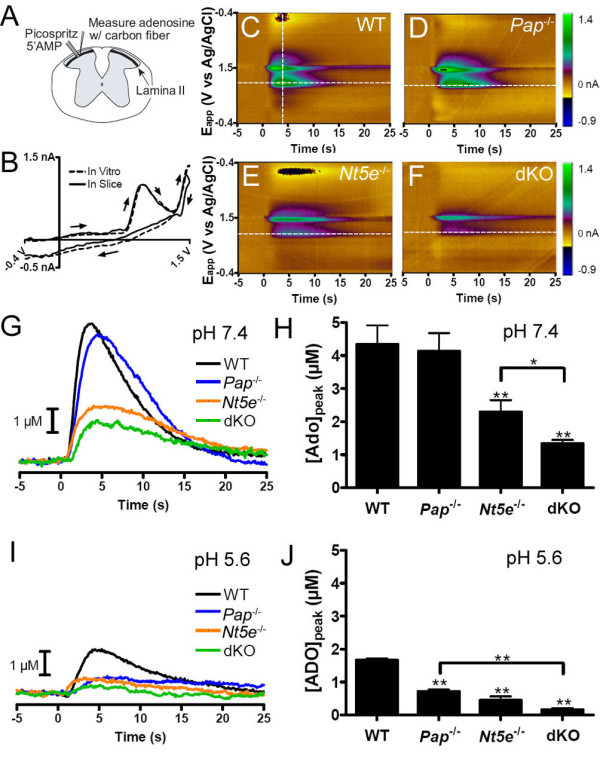
**Adenosine production is impaired in spinal nociceptive circuits of *Pap***^***-/-***^***, Nt5e***^***-/-***^** and dKO mice**. FSCV was used to measure adenosine production at subsecond resolution. (A) Illustration depicting the placement of the carbon fiber microelectrode and the micropipette for pressure ejection of AMP into lamina II (transverse section shown to highlight anatomy; however, sagittal sections were used for these experiments). (B) Normalized cyclic voltammograms obtained for adenosine in physiological buffer (*in vitro*, dashed trace) and during pressure ejection of AMP into lamina II of a WT slice (solid trace; taken from the dashed vertical line in panel C). (C-F) Color plots for the 1 s pressure ejection of 100 μM AMP into lamina II of (C) WT, (D) *Pap*^-/-^, (E) *Nt5e*^-/-^, and (F) dKO slices at pH 7.4. (G) Current extracted at 1.0 V from the dashed horizontal lines in (C-F), converted to concentration of adenosine and plotted versus time. (H) Peak adenosine production following pressure ejection of AMP into lamina II at pH 7.4 (n = 5 for each genotype). (I) Current extracted at 1.0 V from representative experiments performed at pH 5.6. (J) Peak adenosine production following pressure ejection of AMP into lamina II at pH 5.6 (n = 5 for each genotype). (H, J) One way ANOVAs with post-hoc tests were used to compare each genotype to WT and to compare between genotypes. *P < 0.05, **P < 0.005. Data presented as means ± s.e.m.

It is currently unknown how rapidly ectonucleotidases generate adenosine in any tissue or cell of the body. This lack of knowledge reflects technical limitations in existing biochemical and biosensor based methods, all of which sample adenosine levels over minutes to hours [18,19,23,24,27]. And, enzyme histochemistry detects inorganic phosphate following AMP hydrolysis, not adenosine. As such, enzyme histochemistry is an indirect way to measure adenosine production. Thus, to directly quantify the speed and magnitude of adenosine production in the anatomical microdomain where PAP and NT5E are located (axon terminals in lamina II) we used FSCV--an electrochemical technique that can detect adenosine at subsecond resolution without interference from metabolites (like inosine) [23]. This technique can also distinguish adenosine from nucleotides (like ATP, ADP, AMP) by an oxidation potential at 1.0 V that is unique to adenosine. To perform these studies, we pressure ejected AMP (100 μM) into lamina II of sagittal spinal cord slices taken from WT, *Pap*^*-/-*^*, Nt5e*^*-/-*^ and dKO mice while measuring adenosine with a carbon-fiber microelectrode (Figure 3A). Characteristic cyclic voltammograms for adenosine with peaks at 1.0 and 1.5 V (Figure 3B; *in vitro*) were observed after AMP was ejected into lamina II of WT slices (Figure 3B, C; in slice) [23]. Moreover, the oxidation current at 1.0 V peaked 2-3 s after the oxidation current at 1.5 V (Figure 3C), indicative of enzyme-dependent hydrolysis of AMP to adenosine. The 1.0 V peak was not observed when AMP was ejected into buffer, an agar block or outside lamina II (data not shown), confirming the 1.0 V peak is unique to adenosine.

In WT slices, adenosine levels peaked within ~4 s at a concentration of 4.34 ± 0.57 μM at pH 7.4 (Figure 3C, G). Adenosine production and peak levels (4.13 ± 0.55 μM) were not impaired in *Pap*^*-/-*^ slices at pH 7.4 (Figure 3D, G, H), consistent with no visible reduction in AMP hydrolysis at this pH (Figure 1F). However, adenosine production and peak levels were significantly impaired in *Pap*^*-/-*^ slices at pH 5.6 compared to WT slices (0.72 ± 0.05 μM verses 1.66 ± 0.06 μM, respectively, Figure 3I, J). In addition, adenosine production and peak levels were significantly impaired in *Nt5e*^-/-^ and dKO slices at neutral and acidic pH (Figure 3E-J). Notably, peak adenosine levels at pH 7.4 were significantly lower in dKO slices than in *Nt5e*^-/-^ slices (1.34 ± 0.11 μM verses 2.30 ± 0.36 μM, respectively), unmasking a contribution of PAP to adenosine production at neutral pH, similar to histochemical studies with dKO tissues (Figure 1H). Lastly, at pH 5.6, almost no adenosine was produced after pressure ejecting AMP into lamina II of dKO slices (Figure 3I, J). Taken together, these direct measures of adenosine with gene knockout mice indicate that PAP and NT5E rapidly generate adenosine at neutral and acidic pH and together are the main adenosine-generating ectonucleotidases in spinal nociceptive circuits.

Remarkably, some adenosine was still produced in dKO spinal slices at pH 7.4 (Figure 3H), suggesting there is at least one more adenosine-generating enzyme (see Discussion). The presence of this third enzyme could not have been anticipated without first studying *Pap/Nt5e *dKO mice.

### Adenosine transients occur spontaneously and are generated by PAP and NT5E

**Figure 4:**
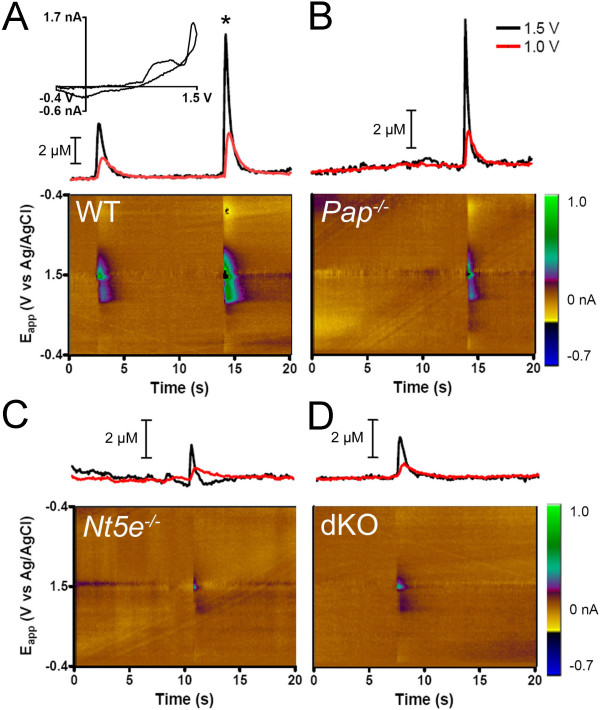
**Spontaneous adenosine transients detected in lamina II by FSCV**. (A-D) Concentration versus time traces (top) and color plots (bottom) in slices (lamina II) from (A) WT, (B) *Pap*^*-/-*^, (C) *Nt5e*^*-/-*^, and (D) dKO mice. Scale bars are for the 1.0 V traces. (A-inset) Cyclic voltammogram for the transient marked with an asterisk. Voltammograms had the signature shape for adenosine, as shown in Figure 3B. The abrupt vertical lines in the color plots are due to a change in double-layer capacitance when fast events occur at the electrode surface, as seen previously [56,57].

While performing FSCV studies, we unexpectedly observed "adenosine transients"--spontaneous and transient increases in adenosine concentration--every time a freshly-polished carbon-fiber microelectrode was placed in lamina II of WT slices (Figure 4A). We also detected transients in some, but not all, slices from *Pap*^*-/-*^*, Nt5e*^*-/-*^*, *and dKO mice (Figure 4B-D). We did not see these transients if microelectrodes were re-used without being re-polished, suggesting debris on the tip interferes with detection of these transients. The peaks at 1.0 V and 1.5 V in color plots and cyclic voltammograms (Figure 4) confirmed that these transients contained adenosine. Transient release was observed at a low frequency (0.35 ± 0.04 events/min) in all WT slices (Table 1; with 2.5 mM extracellular calcium).

**Table 1:** Summary of adenosine transient events in spinal cord lamina II.

Genotype	**[Ca**^**2+**^**] (mM)**	**[Ado]**_**peak**_**(nM)**	Frequency (events/min)	**Transient Time (s)**^**#**^
WT (n = 19)	2.5	570 ± 50	0.35 ± 0.04	1.5 ± 0.1
WT (n = 5)	0.0	410 ± 50	0.06 ± 0.03**	1.8 ± 0.3
*Pap*^*-/-*^ (n = 10)	2.5	690 ± 150	0.14 ± 0.04*	1.5 ± 0.1
*Nt5e*^*-/-*^ (n = 10)	2.5	360 ± 20	0.14 ± 0.07*	1.5 ± 0.1
dKO (n = 16)	2.5	380 ± 40	0.10 ± 0.03**	1.2 ± 0.1

Statistics: One-way ANOVA (*P *< 0.05) followed by Newman-Keuls multiple comparison post-hoc test to compare all genotypes relative to WT (2.5 mM Ca^2+^); *P < 0.05, **P < 0.005, ***P < 0.0005.

^#^ Transient time was measured from the onset of the transient until the signal decayed to baseline.

To ascertain if these events were Ca^2+^ dependent, we chelated intracellular Ca^2+^ by pre-incubating slices in 10 μM BAPTA-AM for 45 minutes and removed extracellular Ca^2+^ from the bath. Under these conditions, adenosine transients were observed in four of the five slices, at a frequency (0.06 ± 0.03 events/min) that was significantly less than when calcium was present in the bath (Table 1). PAP and NT5E do not require divalent cations for activity (see histochemistry buffer in Methods), so this reduction was not likely due to inhibition of PAP or NT5E.

**Figure 5:**
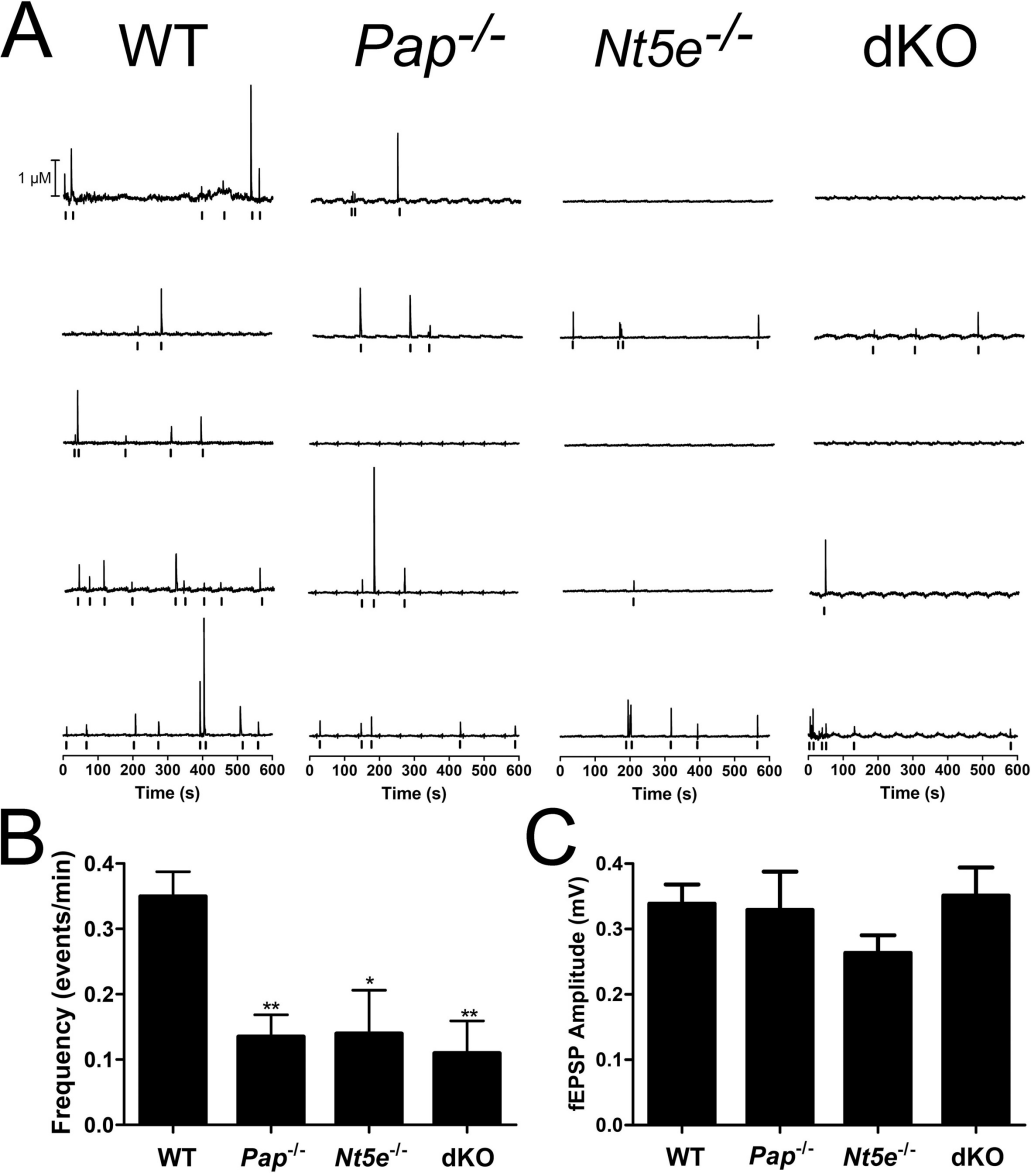
**Spontaneous adenosine transients in lamina II are reduced in frequency in *Pap***^***-/-***^***, Nt5e***^***-/-***^** and dKO mice**. (A) Representative traces from each genotype showing adenosine concentration versus time, calculated from FSCV currents measured at 1.5 V. Traces were background subtracted every 60 s and baseline corrected to compensate for electrode drift over time (which creates a visible 60 s oscillation in the baseline). Raster plots below each trace mark each adenosine transient. Adenosine transients were considered to be events if the peak at 1.0 V was more than five times the standard deviation of the noise. (B) Adenosine transient frequency in WT (0.35 ± 0.04 events/min; n = 19 slices), *Pap*^-/-^ (0.14 ± 0.04 events/min; n = 10 slices), *Nt5e*^-/-^ (0.14 ± 0.07 events/min; n = 10 slices), and dKO (0.11 ± 0.05 events/min; n = 16 slices) mice. One-way ANOVAs with Bonferroni's post-hoc tests were used to compare each genotype to WT and to compare between genotypes. *P < 0.05, **P < 0.005. (C) Baseline fEPSP amplitude in WT, *Pap*^-/-^, *Nt5e*^-/-^, and dKO mice (n = 5/genotype). There were no significant differences between WT and mutant mice. Data presented as means ± s.e.m.

Next, to determine if PAP and NT5E generate adenosine transients, electrodes were placed into *Pap*^-/-^, *Nt5e*^-/-^, and dKO slices and up to 20 consecutive one-minute intervals were recorded, background subtracted and corrected to account for background drift of the electrode over time (Figure 5A, experiments with WT slices were interleaved with knockout slices). Unlike WT slices where transients were observed in every (19/19) slice, spontaneous events were observed in a subset of slices from *Pap*^-/-^ (8/10 slices), *Nt5e*^-/-^ (6/10 slices) and dKO (9/16 slices) mice. Furthermore, in slices from *Pap*^*-/-*^, *Nt5e*^*-/-*^ and dKO mice where transients were observed, the frequency was significantly lower than in WT slices (Figure 5B). This reduction is unlikely to be due to deficits in synaptic transmission since evoked field excitatory postsynaptic potential (fEPSP) amplitudes were not significantly different between WT and mutant backgrounds (Figure 5C). Collectively, these findings (summarized in Table 1) indicate PAP and NT5E generate a majority of all adenosine transients, presumably through hydrolysis of nucleotides that are released by neurons and/or glia (see Discussion). Moreover, the observation that transient frequency can be reduced in single and dKO mice suggests these transients can be dynamically modulated by manipulating ectonucleotidase activity.

### PAP and NT5E generate an A_1_R-dependent inhibitory tone in nociceptive circuits

**Figure 6:**
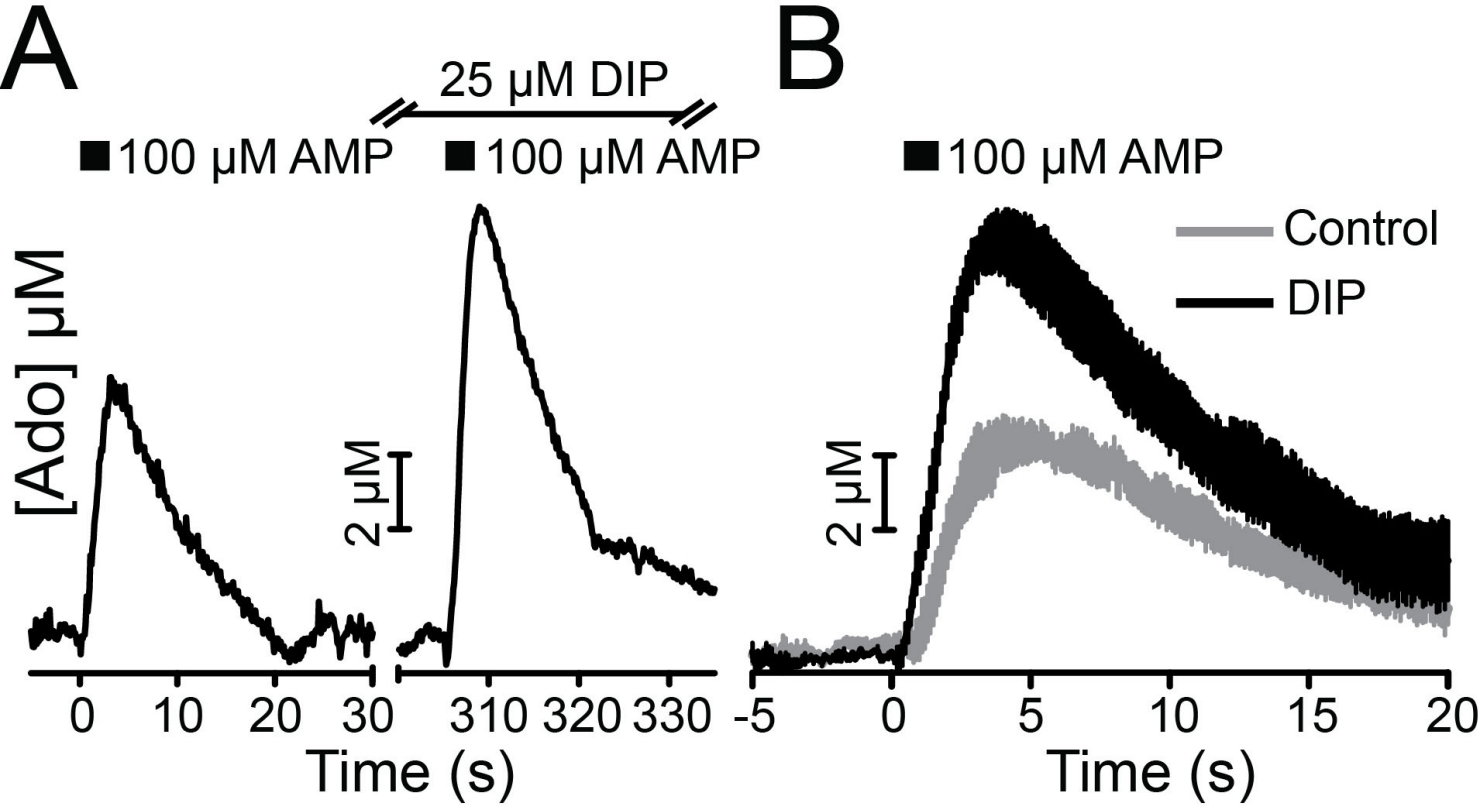
**A nucleoside transport inhibitor promotes the buildup of ectonucleotidase-generated adenosine**. (A) AMP was pressure ejected into lamina II of WT spinal cord slices in the absence or presence of DIP. FSCV was used to measure extracellular adenosine. (B) Average responses ± s.e.m., from n = 3 slices. DIP significantly increased the concentration of extracellular adenosine from 0.7 to 8 s following AMP addition (*P *< 0.005 by one-way ANOVA).

**Figure 7:**
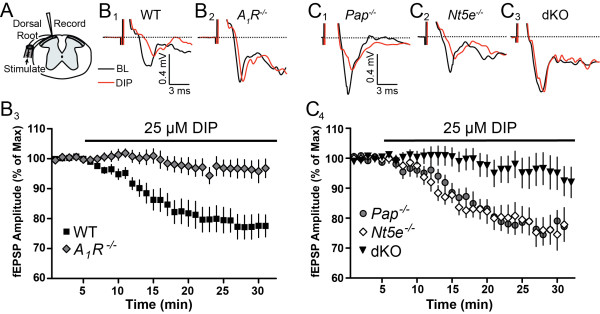
**Dipyridamole, an adenosine transport blocker, gradually inhibits excitatory synaptic transmission in lamina II of WT mice but not A**_**1**_**R or dKO mice**. (A) Illustration depicting the placement of the suction electrode to stimulate fEPSPs and the recording electrode in lamina II. (B_1_-B_2_) Representative Aδ fEPSPs in WT and *A*_*1*_*R*^*-/-*^ spinal cord slices before (BL; black) and 25 min after (red) addition of DIP (25 μM) to the perfusate. (B_3_) Normalized fEPSP amplitude in WT and *A*_*1*_*R*^*-/-*^ spinal cord slices (n = 15, and 14 respectively, *P *< 0.001 by two-way ANOVA and Bonferroni's post-hoc tests). (C_1_-C_3_) Representative Aδ fEPSPs in *Pap*^*-/-*^*, Nt5e*^*-/-*^, and dKO spinal cord slices before (black) and after (red) addition of DIP (25 μM). (C_4_) Normalized fEPSP amplitude in *Pap*^*-/-*^*, Nt5e*^*-/-*^*, *and dKO spinal cord slices (n = 13, 12 and 16 respectively). The normalized fEPSP amplitude after DIP addition was significantly larger in dKO slices when compared to WT (*P *< 0.001 by two-way ANOVA and Bonferroni's post-hoc tests). There were no significant differences between WT and *Pap*^*-/-*^ or WT and *Nt5e*^*-/-*^ fEPSP amplitudes after DIP addition. (B_3_, C_4_) Data are presented as means ± s.e.m.

Adenosine has a tonic inhibitory effect on excitatory neurotransmission in the spinal cord and brain [6,15,16,28], although the molecular origins of this inhibitory tone are unknown. Since PAP and NT5E rapidly generate adenosine from nucleotides and deletion of PAP and NT5E reduced the frequency of adenosine transients, we hypothesized that PAP and NT5E might generate the inhibitory adenosine tone in nociceptive circuits. To test this hypothesis, we measured evoked field excitatory postsynaptic potential (fEPSP) amplitude in lamina II in the absence and presence of DIP with the rationale that endogenously generated adenosine will build up in the presence of DIP and inhibit excitatory neurotransmission. Indeed, DIP significantly elevated extracellular adenosine levels for an extended time period when AMP was pressure ejected into lamina II (Figure 6A, B), indicating DIP promotes the buildup of ectonucleotidase-generated adenosine outside cells. In the absence of DIP, dorsal root stimulation at very low frequency (0.1 Hz) evoked a potential with rapid onset and clearly discernable peak in WT and *A*_*1*_*R*^-/-^ slices (Figure 7A, 7B_1_-B_2_, black trace), classified as an Aδ-fiber waveform [29]. After application of DIP, this Aδ-fiber fEPSP significantly decreased in amplitude from baseline in WT slices but not in *A*_*1*_*R*^-/-^ slices (Figure 7B_1_-B_3_, red trace).

To determine if this gradually developing A_1_R-dependent inhibition was ectonucleotidase-dependent, we tested slices from *Pap*^*-/-*^*, Nt5e*^*-/-*^ and dKO mice (interleaved with the WT and *A*_*1*_*R*^-/-^ slices above). While fEPSP amplitude was equally reduced (inhibited) in slices from WT, *Pap*^*-/-*^ and *Nt5e*^*-/-*^ mice following DIP addition (Figure 7C_1_, C_2_, C_4_), fEPSP amplitude was not significantly reduced when DIP was added to dKO slices (Figure 7C_3_, C_4_). These results suggest that PAP and NT5E individually generate sufficient amounts of adenosine to inhibit excitatory neurotransmission; however, when both enzymes are deleted, not enough adenosine builds up over the time scale of these experiments to significantly activate A_1_R and inhibit excitatory neurotransmission. Like the *in vivo *experiments with DIP and ITU (where A_1_R activity is integrated over an extended time period), these experiments revealed pronounced deficits in dKO mice that were not apparent when PAP or NT5E were individually deleted.

### Nociception is enhanced in single and dKO mice

**Figure 8:**
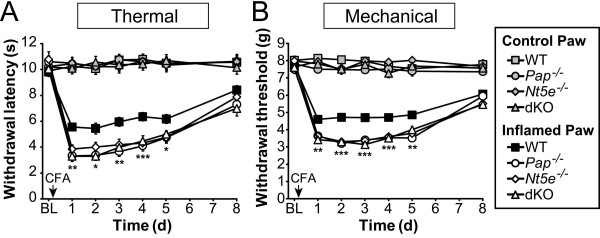
**Inflammatory heat hyperalgesia and mechanical allodynia are enhanced in *Pap***^***-/-***^***, Nt5e***^***-/-***^** and dKO mice**. (A, B) WT, *Pap*^*-/-*^*, Nt5e*^*-/-*^*, *and dKO mice were tested for (A) thermal and (B) mechanical sensitivity before (BL) and following injection of CFA (arrow) into one hindpaw. The contralateral hindpaw served as control. All data are presented as means ± s.e.m. A repeated measures two-way ANOVA (*P *< 0.0005 for all genotypes) with Bonferroni's post-hoc test was used to compare WT to *Pap*^*-/-*^*, Nt5e*^*-/-*^*, *and dKO mice (n = 9-10 mice per group). *P < 0.05; **P < 0.005, ***P < 0.0005. There were no significant differences between dKO and *Pap*^*-/-*^ or dKO and *Nt5e*^*-/-*^ mice.

Noxious thermal and mechanical stimuli are sensed by nociceptors that excite postsynaptic lamina II neurons in spinal cord. Since the tonic inhibitory effects of adenosine were impaired in lamina II of dKO mice, we hypothesized that deletion of *Pap *and *Nt5e *might enhance noxious thermal and/or mechanical sensitivity. To test this hypothesis, we studied WT, *Pap*^*-/-*^*, Nt5e*^*-/*^^-^, and dKO mice using behavioral models of acute and chronic pain. We found that *Pap*^*-/-*^, *Nt5e*^*-/-*^ and dKO mice had no significant differences in mechanical sensitivity (electronic Von Frey) and no significant differences in two measures of noxious thermal sensitivity (Hargreaves test and hotplate test at 52°C) (Table 2). At a higher hotplate temperature (55°C), withdrawal latency was significantly enhanced (shorter latency) in dKO mice. All three mutant genotypes showed enhanced thermal nociception in the tail immersion assay at 49.0°C while *Nt5e*^-/-^ and dKO mice showed enhanced thermal nociception at 46.5°C. Likewise, thermal hyperalgesia and mechanical allodynia were significantly and equally enhanced in *Pap*^*-/-*^, *Nt5e*^-/-^ and dKO mice (relative to WT) following inflammation with complete Freund's adjuvant (CFA) (Figure 8A, B). Collectively, these experiments with naïve (non-sensitized) and sensitized animals suggest that PAP and NT5E tonically inhibit nociception *in vivo*. Given that dKO mice showed more pronounced physiological and behavioral deficits when compared to single knockouts in several of the experiments above, it is unclear why dKO mice did not show more pronounced behavioral deficits in these acute or chronic pain behavioral assays. Although as previously discussed [10], this could reflect a number of possibilities, with the most likely being biologically imposed floor effects--mice may be unable to sense stimuli and/or withdrawal their paws any faster.

**Table 2:** Acute mechanical and thermal sensitivity

Behavioral Assay	WT	*Pap*^*-/-*^	*Nt5e*^*-/-*^	dKO
	* Withdrawal threshold*:			
Electronic von Frey	7.8 ± 0.3 g	7.8 ± 0.4 g	8.0 ± 0.2 g	8.0 ± 0.2 g
	*Withdrawal latency*:			
Radiant heating of hindpaw (Hargreaves Method)	9.9 ± 0.4 s	10.2 ± 0.4 s	10.6 ± 0.4 s	10.2 ± 0.4 s
Tail immersion at 46.5°C	16.9 ± 1.2 s	14.3 ± 0.7 s	12.6 ± 0.6 s**	13.1 ± 1.0 s*
Tail immersion at 49.0°C	7.2 ± 0.7 s	4.7 ± 0.2 s***	4.0 ± 0.2 s***	4.7 ± 0.3 s**
Hot plate at 52°C	33.5 ± 1.7 s	37.7 ± 2.6 s	29.6 ± 1.5 s	31.1 ± 2.2 s
Hot plate at 55°C	16.2 ± 1.1 s	13.5 ± 0.5 s	13.5 ± 1.1 s	12.7 ± 0.8 s*

Data are expressed as means ± s.e.m. One-way ANOVAs were performed with Bonferroni's post hoc test to compare each genotype to WT. For post hoc tests, *P < 0.05, **P < 0.005, ***P < 0.0005. n = 10 male mice tested per genotype.

## Discussion

Nucleotides cause pain by exciting and sensitizing nociceptive neurons. While the duration of nucleotide signaling can be limited by purinergic receptor desensitization and nucleotide hydrolysis, it was unknown how rapidly specific ectonucleotidases break down nucleotides in any region of the nervous system. The speed at which hydrolysis occurs could affect the balance between the excitatory effects of nucleotides and the inhibitory effects of adenosine. While we previously found that PAP and NT5E were co-expressed in nociceptive neurons and could hydrolyze AMP [10,11], we did not previously show that either enzyme could generate adenosine directly in nociceptive circuits or how fast these enzymes generate adenosine. Moreover, since our studies were focused on *Pap *and *Nt5e *single knockout mice, we could not predict *a priori *if these enzymes had redundant or non-redundant functions.

To resolve these issues, we generated *Pap/Nt5e *dKO mice--the first double knockout of any pair of ectonucleotidases. Our analysis revealed that PAP and NT5E together generate the majority of all adenosine from AMP in nociceptive axon terminals. FSCV allowed us to monitor the speed at which these ectonucleotidases generate adenosine. And, the small FSCV probes allowed us to selectively monitor adenosine production in the spinal microdomain where these enzymes are located. To our surprise, we found that PAP and NT5E very rapidly generate adenosine from pressure ejected AMP and from endogenously released nucleotides.

Our conclusions are supported by an array of *in vitro *and *in vivo *experiments. Indeed, we found that dKO animals had pronounced deficits in hydrolyzing AMP to adenosine, AMP → adenosine-dependent antinociception, and AMP → adenosine-dependent synaptic inhibition. While a pH preference for these enzymes was evident in histochemical and FSCV experiments, both enzymes contribute additively to nucleotide hydrolysis under physiologically relevant *in vivo *conditions (Figure 2). PAP and NT5E are likely to encounter wide extracellular pH fluctuations under normal physiological and pathological conditions. For example, excitatory synapses become transiently acidified following glutamate release [30], synaptic vesicle pH can reach pH 5.6 [31], and pathological conditions lead to prolonged acidification and nucleotide release [32].

Our experiments also revealed that at least one more adenosine-generating ectonucleotidase is present in nociceptive circuits. This finding could not have been predicted from studying *Pap *or *Nt5e *single knockouts, and suggests nociceptive circuits have a highly redundant molecular apparatus for eliminating nucleotides while simultaneously generating adenosine. While definitive molecular identification of this third ectonucleotidases is beyond the scope of our present study, our data do provide genetic and biochemical clues as to what this additional ectonucleotidase(s) might be. AMP hydrolysis and adenosine production were largely eliminated in lamina II at pH 5.6 in dKO mice, making it unlikely this enzyme is another acid phosphatase. However, this enzyme could have a neutral to alkaline pH optimum given that adenosine is produced at pH 7.4 (albeit at levels that are only ~30% of WT levels; Figure 2H). Alkaline phosphatases are present in the spinal cord, have such a pH optimum and can hydrolyze ATP, ADP and AMP extracellularly [7,33]. Whether alkaline phosphatases (of which there are four functional genes in the mouse) or other ectonucleotidases contribute to residual adenosine production in spinal nociceptive circuits remains to be determined.

FSCV allowed us to measure rapid changes in adenosine concentration in a micron-sized region of spinal cord. The temporal and spatial resolution of this method far surpasses all previously used methods for measuring adenosine (including direct approaches like enzyme bioprobes or microdialysis coupled with HPLC and indirect approaches like quantifying signaling downstream of adenosine receptors) [18,19,23,24,27,34]. Unexpectedly, we detected spontaneous adenosine transients in spinal lamina II, the termination zone for nociceptive afferents. This is the first time adenosine transients were detected in any region of the nervous system. The duration of these events was remarkably short (lasting 1.5 ± 0.1 s on average in WT mice; Table 1), suggesting adenosine is rapidly generated/released within lamina II, precisely where PAP and NT5E are found [10,11]. Transients were detected in all WT slices but only ~50% of all *Pap*^*-/-*^, *Nt5e*^-/-^ and dKO slices. And, within the subset of *Pap*^*-/-*^, *Nt5e*^-/-^ and dKO slices where transients were observed, the frequency of adenosine transients was reduced by more than half. These data strongly suggest that PAP and NT5E generate adenosine transients in lamina II by rapidly hydrolyzing endogenously released nucleotides. Rapid generation of adenosine is consistent with ectonucleotidases being in close proximity to nucleotide release sites [35].

Nucleotides like ATP are released from several cell types by point-source burst release, which occurs spontaneously at a low frequency (0.38 ± 0.18 bursts/min in rat astrocytes) [1]. Intriguingly, adenosine transients occur at a similar frequency (0.35 ± 0.04 events/min; Table 1), possibly reflecting the pulsatile availability of nucleotide substrate. Several distinct mechanisms of ATP release have been reported in neurons, glia and other cell types, including from vesicles (exocytosis), connexin 43 hemichannels, P2X7 ion channels and volume activated anion channels [36-43]. Many of these mechanisms are kinetically fast and Ca^2+^ dependent. Future studies will be needed to determine which cells release ATP and which molecular mechanisms underlie nucleotide release in nociceptive circuits.

Our data also suggests it might be possible to dynamically regulate adenosine transient frequency through physiological and genetic factors. Indeed, we found adenosine transients were reduced in frequency in WT slices under zero Ca^2+^ conditions and in dKO slices; however, transients did not disappear (Table 1). Similarly, capsaicin-evoked adenosine release from synaptosomes (isolated from dorsal spinal cord) was reduced in Ca^2+^-free conditions and when nonselective ectonucleotidase inhibitors were used [19]. Since adenosine transients were not eliminated in dKO mice, there must be additional mechanisms underlying their generation. These mechanisms could include: 1.) hydrolysis of extracellular nucleotides by ectonucleotidase(s) other than PAP and NT5E and/or 2.) direct release of adenosine from cells, such as by exocytosis or reverse transport through equilibrative nucleoside transporters [17,19].

### Physiological significance of pulsatile adenosine

Our physiological and behavioral data suggest that ectonucleotidases, and the adenosine transients they generate, endow nociceptive circuits with a way to transform pulsatile or sustained nucleotide release into an inhibitory adenosinergic signal. Notably, we found that transients were generated in all WT slices but only in ~50% of all *Pap*^*-/-*^, *Nt5e*^-/-^ and dKO slices, suggesting more "failures" when ectonucleotidases are eliminated. Moreover, the frequency of these events was significantly reduced in slices from all three mutant genotypes. The failure to generate transients, combined with reduced transient frequency likely explains why excitatory neurotransmission was not inhibited after treating dKO slices with DIP (Figure 7). These data suggest that PAP and NT5E generate adenosine endogenously at a level that is sufficient to activate A_1_R and inhibit excitatory neurotransmission in lamina II. However, in the absence of these enzymes, less adenosine is tonically produced, causing unimpeded excitatory neurotransmission and enhanced nociceptive responses.

In addition, adenosine transients and the enzymes that generate these transients appear to be biochemically "tuned" to dynamically activate A_1_R. In particular, transients have a peak adenosine concentration of 530 ± 60 nM as measured by FSCV (Table 1), and could be slightly higher given that analyte concentration is distance dependent with respect to its point of release/production [44]. Extracellular adenosine concentration in hippocampus was similarly ~300-1500 nM [28]. This concentration range is remarkably similar to the concentration range that half-maximally stimulates A_1_R (760 nM-3.1 μM) [45-47]. Since the peak adenosine concentration of each transient falls within the most sensitive region of the A_1_R dose-response curve, small increases or decreases in transient frequency, especially when integrated over time, could have profound effects on A_1_R signaling. Indeed, excitatory potentials were inhibited in an A_1_R-dependent manner only when PAP and NT5E were both deleted (Figure 7), suggesting both enzymes generate more adenosine over time than either enzyme alone. Lastly, ectonucleotidases can "channel" adenosine directly to A_1_R [48], which could further maximize the signaling effects of enzymatically produced adenosine.

## Conclusions

Our data indicate that PAP and NT5E rapidly transform nucleotides in nociceptive circuits into an inhibitory adenosine tone that is pulsatile and subject to frequency modulation. Ectonucleotidases are present and functional throughout the brain [49]. These enzymes could thus dynamically modulate diverse physiological and pathological processes--like sleep, synaptic plasticity and striatal motor function--that are under tonic adenosine control [50,51].

## Methods

### Animals

All procedures and behavioral experiments involving vertebrate animals were approved by the Institutional Animal Care and Use Committee at the University of North Carolina at Chapel Hill. Mice were raised under a 12:12 light:dark cycle and fed Prolab RMH 3000 (LabDiet) *ad libitum*. C57BL/6 mice were purchased from Jackson Laboratories or bred in house from C57BL/6J stock. *Pap*^*-/-*^ (Vihko et al., abstract from Proceedings of the AACR, 2005, 96^th^ Annual Meeting, Anaheim, CA) [11]*, Nt5e*^*-/-*^[52] and *A*_*1*_*R*^*-/-*^[12] mice were backcrossed to C57BL/6J mice for > 10 generations. dKO mice were generated by breeding backcrossed *Nt5e*^*-/-*^ and *Pap*^*-/-*^ mice.

### Enzyme histochemistry

AMP histochemistry was performed as previously described [10,11]. Briefly, adult male mice 6-12 weeks of age were killed by decapitation. Lumbar spinal cord and DRG were dissected and immersion fixed in 4% paraformaldehyde in 0.1 M phosphate buffer at pH 7.4 (8 and 4 hours respectively). Tissue was cryoprotected in 30% sucrose in 0.1 M phosphate buffer pH 7.3 at 4°C for at least 24 hours. DRG and spinal cord were sectioned (20 μm and 30 μm thick, respectively) on a cryostat and were collected on Superfrost Plus slides (DRG) or as free-floating sections (spinal cord). Enzyme histochemistry was performed using 1 or 6 mM AMP as substrate in Tris-maleate buffer at pH 5.6 or pH 7.0 with 2.4 mM lead nitrate. Staining intensity was quantified using ImageJ as follows: a rectangle was drawn over lamina II, then mean grey scale (a measure of pixel intensity) was determined from multiple (7-10) images from each genotype and averaged. We then took the inverse of the mean pixel intensity and multiplied by 1000 so that larger numbers corresponded to darker staining.

### Behavior

Male mice, 2-4 months old, were acclimated to the testing room, equipment and experimenter for 1-3 days before behavioral testing. The experiments were carried out during the light phase and the experimenter was blind to the genotype during testing. The Hargreaves method was used to test noxious thermal sensitivity. One measurement was taken from each paw to determine withdrawal latency. The radiant heat source cutoff time was 20 s. The tail-immersion assay and hot plate test were carried out as previously described [10]. Mechanical sensitivity was measured using an electronic von Frey apparatus (IITC) as described previously [11]. Three measurements were taken from each paw (separated at 10 min. intervals) then averaged to determine paw withdrawal threshold in grams. Complete Freund's adjuvant (20 μl CFA; MP Biomedicals) was injected into one hindpaw, centrally beneath glabrous skin with a 30G needle. Adenosine 5'-monophosphate (AMP; 80 mM stock, Fluka 01930) and 5-iodotubericidin (ITU, 25 mM stock, Biomol, EI-293) were dissolved in 0.9% saline and DMSO, respectively, and were then diluted in 0.9% saline before use. Dipyridamole (DIP, 4 mM stock; Sigma D9766) was dissolved in 0.9% saline slightly acidified with HCl as described [25]. All drugs were intrathecally injected (5 μl) into unanethetized mice using the direct lumbar puncture method.

### Slice preparation

Transverse (800-900 μm, used in field recordings) and sagittal (400 μm, used in FSCV experiments) spinal cord slices were prepared as previously described from 1-2 month old mice [53]. Briefly, mice were anesthetized with urethane (1.5 g/kg, i.p.) and the lumbar spinal cord was quickly dissected and sliced on a Vibratome 3000EP at 4°C. The dissection buffer contained the following (in mM): 87 NaCl, 2.5 KCl, 1.25 NaH_2_PO_4_, 25 NaHCO_3_, 75 sucrose, 10 glucose, 1.5 ascorbic acid, 0.5 CaCl_2_, 7 MgCl_2_. The slices were then incubated at 37°C for 45 minutes and then at room temperature in regular artificial cerebrospinal fluid (ACSF) that contained (in mM): 125 NaCl, 2.5 KCl, 1.25 NaH_2_PO_4_, 26 NaHCO_3_, 25 glucose, 2.5 CaCl_2_, 1.5 MgCl_2_. For cyclic voltammetry experiments, the slices were incubated for one hour or less before the experiments were carried out. All solutions were bubbled with 95% O_2_/5% CO_2_ for the duration of the dissection and incubation steps.

### Fast-scan cyclic voltammetry of adenosine

Disk-shaped carbon-fiber microelectrodes were fabricated as previously described [54]. Briefly, a single 6 μm diameter T-650 carbon fiber (Thornell, Amoco Corp. Greenville, SC) was aspirated into a glass capillary (1.2 mm × 0.68 mm, AM Systems, Carlsborg, WA) which was then sealed on a vertical pipette puller (model PE-21, Narishige Group, Japan). Electrodes were then cut with a surgical blade, sealed with epoxy, and polished at 30° on a K. T. Brown micro-pipette beveller (model BV-10, Sutter Instruments, Novato, CA). Before use, electrodes were soaked in a mixture of isopropanol and activated carbon for at least 20 minutes [55]. To detect adenosine, the electrode's potential was held at -0.4 V between each scan, and was ramped from -0.4 V to 1.5 V at a scan rate of 400 V/s every 100 ms. The peak at 1.0 V was used to quantify the peak concentrations of adenosine because it is unique to adenosine and not observed with ATP, ADP or AMP [23]. An Ag/AgCl reference electrode was used for all experiments.

FSCV data was collected using a custom LABVIEW program, Tar Heel CV. Two computer interface boards (National Instruments PCI 6052 and PCI 6711, Austin, TX) were used to apply the triangular waveform, digitize the resulting current, and to control pressure ejection synchronization with the waveform application through a homemade breakout box. A Chem-Clamp Voltammeter (Dagan Corporation, Minneapolis, MN) was used to control the electrode potential and measure the resulting current, which was filtered with a built-in analog low pass Bessel filter at 10 kHz. To make electrical contact with the head stage, a stainless steel wire was inserted into the back of the microelectrode which was backfilled with an electrolyte mixture of 4 M potassium acetate and 0.15 M potassium chloride.

FSCV data were viewed in the form of color plots with sequentially stacked cyclic voltammograms shown over time (abscissa), plotted against the electrode potential displayed on the ordinate where the switching potential (1.5 V) is in the middle. The current is displayed in false color, with oxidative currents being shown in green and reductive currents being shown in blue and black. From these plots, the current at a specific potential can be plotted against time to examine how the adenosine concentration changes. These current traces are converted to concentration from calibrations performed in a flow injection apparatus which allows for a bolus of adenosine ranging in concentration from 1-10 μM or 10 μM AMP to be introduced to the electrode surface.

To detect the production of adenosine by PAP and NT5E, 100 μM AMP was pressure ejected five times at 5 minute intervals using a Picospritzer^®^ III (Parker Instrumentation, Pinebrook, NJ) for 1 s at 20 psi from a micropipette inserted into sagittal slices, approximately 100 μm from the carbon fiber microelectrode. To minimize tissue damage, electrodes were inserted at 30° from the slice surface with the disk facing down, approximately 50 μm below the surface of the slice. AMP solutions were freshly prepared for each experiment to overcome the possible confound of degradation into adenosine. Furthermore, the absence of adenosine in the pressure ejection pipette was confirmed before and after each experiment by ejections onto the electrode above the slice (confirmed by the absence of current at 1.0 V). For experiments carried out at pH 5.6, five consecutive ejections at 5 minute intervals were performed 10 minutes after application of pH 5.6 buffer. The buffer used for experiments at pH 5.6 contained in mM: 10 Tris Maleate, 140 NaCl, 4 KCl, 2 MgCl_2_, 2 CaCl_2_, 5 Glucose, and pH adjusted to 5.6 with NaOH. When detecting spontaneous adenosine transients, the carbon fiber was inserted into the slice without any stimuli applied to the slice. For adenosine assignment, each event had a distinguishable cyclic voltammogram with a current peak at 1.0 V that was more than 5 times the standard deviation of the baseline noise.

### Field potential recordings

Transverse slices containing one dorsal root were placed in the chamber and the root was suctioned into a glass electrode in order to stimulate all sensory afferents. Slices were maintained at room temperature and perfused with ACSF at 2 mL/min for the duration of the recordings. Recording electrodes were pulled from borosilicate glass on a Sutter P-97 puller to a tip resistance of 1-2 MΩ when filled with ACSF. Under visualization of the slice under low (5X) magnification, the recording electrode was placed in the medial portion of lamina II (the substantia gelatinosa). The dorsal root was stimulated for 0.5 msec at 5x the intensity needed to evoke a maximal response. The root was stimulated once every 10 seconds (0.1 Hz) and every 6 signals were averaged to give a single point for every minute of recording. The resulting signals were filtered at 1 kHz, amplified 1000 times with a Multiclamp 700B amplifier, captured at 10 kHz with an Axon Digidata 1440A, and analyzed using pClamp 10 software (Molecular Devices). The waveform observed was similar to fEPSPs recorded in transverse rat spinal cord slices [29], with a distinguishable peak that reportedly represents Aδ thinly myelinated afferents, and delayed peaks that were broad and only evoked with high intensity stimulation that likely represent a mixture of unmyelinated c-fiber afferents and polysynaptic activity. DIP (25 mM stock in DMSO) was added to the bath solution after 10 minutes of baseline recording. Only one experiment was carried out per slice. Experiments in which fEPSPs drifted more than 5% during the baseline recording period were not analyzed.

### Statistical Tests

Statistical analysis was carried out using Microsoft Excel, and Graph Pad Prism software.

## Competing interests

The authors declare that they have no competing interests.

## Authors' contributions

SES and PLW designed and carried out experiments, and wrote the paper. NAS carried out mouse behavior experiments. BTB carried out histochemical experiments. TSG and RMW designed FSCV experiments. PV provided the *Pap*^*-/-*^ mice. MJZ conceived the study, designed experiments and wrote the paper. All authors read and approved the final manuscript.
